# Exogeneous Spatial Cueing beyond the Near Periphery: Cueing Effects in a Discrimination Paradigm at Large Eccentricities

**DOI:** 10.3390/vision4010013

**Published:** 2020-02-17

**Authors:** Katharina Weiß

**Affiliations:** Neuro-Cognitive Psychology & Cognitive Interaction Technology Cluster of Excellence (CITEC), Bielefeld University, P.O. Box 100131, D-33501 Bielefeld, Germany; katharina.weiss@ewe.net; Tel.: +49-521-106-6934

**Keywords:** visual attention, far periphery, Posner Cueing

## Abstract

Although visual attention is one of the most thoroughly investigated topics in experimental psychology and vision science, most of this research tends to be restricted to the near periphery. Eccentricities used in attention studies usually do not exceed 20° to 30°, but most studies even make use of considerably smaller maximum eccentricities. Thus, empirical knowledge about attention beyond this range is sparse, probably due to a previous lack of suitable experimental devices to investigate attention in the far periphery. This is currently changing due to the development of temporal high-resolution projectors and head-mounted displays (HMDs) that allow displaying experimental stimuli at far eccentricities. In the present study, visual attention was investigated beyond the near periphery (15°, 30°, 56° Exp. 1) and (15°, 35°, 56° Exp. 2) in a peripheral Posner cueing paradigm using a discrimination task with placeholders. Interestingly, cueing effects were revealed for the whole range of eccentricities although the inhomogeneity of the visual field and its functional subdivisions might lead one to suspect otherwise.

## 1. Introduction

Visual attention has been—and still is—one of the most thoroughly investigated topics in experimental psychology and vision science. This is evident from a large bulk of empirical evidence ([1], for reviews, see [2,3,4,5]) as well as an abundance of theoretical work (e.g., [6,7,8,9]). Nonetheless, knowledge about visual attention is restricted by an often-neglected factor: The size of commercially available computer screens. The majority of attention studies used target eccentricities up to 25° of visual angle [10,11,12], in the rarer cases up to 30° [13,14,15]. To my knowledge, only two studies, Casteau and Smith [16] and Poggel, Strasburger and MacKeben [17] investigated visual attention beyond 30° [16], used eccentricities up to 44°, and [17] even up to 60°. However, mean target eccentricities for most studies on visual attention are much smaller and lie rather between 8°–15°. Thus, empirical knowledge about visual attention beyond 30° in the periphery (near periphery) is sparse. Interestingly, among the minority of attention studies that have used a wider eccentricity range (>20°) are many older studies from the 1970s and earlier. The need for custom-made laboratory equipment before the emergence of personal PCs in the mid-1980s probably allowed investigating a wider eccentricity range more easily e.g., [11,12]. 

Fortunately, the emergence of high-temporal resolution projectors developed for scientific experimentation and virtual reality HMDs allows scientists to tackle the problem of presenting experimental stimuli beyond the near periphery more easily, now. Hence, the present study uses such a projector in combination with a large projection screen to investigate attentional processes not only in the near periphery, but also at more eccentric positions in the far and medium periphery of the visual field. Although the terms near, medium/intermediate, and far periphery are frequently used in empirical literature, they are not applied consistently to specific eccentricity ranges. For instance, while some studies define only eccentricities beyond 60° and larger as far periphery [18,19], other studies already define eccentricities >30°–40° as far or even extreme periphery [16,17]. Therefore, these different parts of the periphery are defined here in accordance with crucial functional subdivisions of the human visual field: Near periphery begins outside foveal vision (>2° from gaze center) and ranges up to 25° because ~25° represents the outer border of a performance plateau for discrimination or recognition tasks (letter recognition thresholds) ([20], p. 27). Thus, medium periphery begins beyond 25°. It ranges until 45° because ~45° constitutes the limit of the effective oculomotor range (EOMR, [21]). The EOMR refers to the eccentricity range that can be reached by an eye movement alone without additional head or body movements. Thus, the EOMR is an important boundary for eye movements. However, most crucially, it might also be an important boundary for attentional processes due to the close link between eye movements and visual attention (e.g., [16]). Hence, the present study defines visual angles above 45° up to the border of the visual field as far periphery. 

The aim of the present study is to determine whether attentional processes in the near periphery show *qualitative* or *quantitative* differences in contrast to processes in medium or far periphery. A qualitative difference would be indicated by expectable facilitative attention effects in the near periphery in contrast to inhibitory or non-existent attention effects in medium and far periphery. However, a quantitative difference would consist only of size changes in facilitative attention effects between these different peripheral regions. Revealing qualitative or quantitative differences between near-, medium-, and far-periphery attention seems plausible due to different reasons, which will now be discussed in more detail.

First, due to the human visual field’s inhomogeneity, the conditions under which attention operates in the near periphery differ considerably from those in the far periphery: Inhomogeneity is evident at different levels of the visual system’s hierarchy along the visual pathway from the retina to the primary visual cortex (V1) and higher visual cortical areas (cf. [6]). Importantly, it is reflected in one of the visual system’s most crucial characteristics—cortical magnification. In V1 and many other cortical areas, a substantially larger ratio of neurons is devoted to processing foveal visual input than to processing visual input from the periphery, although the fovea incorporates only about 2.5° of the visual field. Closely linked to this overrepresentation of the central visual field in many cortical areas is receptive field size. In a great number of cortical areas, receptive field size increases linearly with eccentricity, but density of receptive fields decreases. An interesting exception is the area Prostriata, adjacent to V1 in the calcarine sulcus. Area Prostriata is specialized in fast-moving stimuli in the far periphery ([22,23]), which implies an important role of this area in visual orienting and exogenous attention. In contrast to other cortical areas, Area Prostriata has large receptive fields that are distributed equally over the visual field.

Evidently, inhomogeneity represents a key challenge for visual information processing and basic cognitive functions like object recognition or visual search ([24,25,26,27,28]). The most challenging consequences constitute: (1) Substantial spatial resolution differences across the visual field and (2) a strong susceptibility of peripheral vision to crowding or visual clutter ([29]; for a recent review, see [20]). (1) Spatial resolution—the ability to discriminate between two adjacent points—is highest in the fovea centralis and declines monotonically but dramatically towards the periphery. For instance, at 20° eccentricity, it comprises only a tenth of its foveal value [30]. Thus, for the peripheral visual field, spatial resolution is best in the near periphery, worse in the medium periphery, and worst in the far periphery. Furthermore, within these peripheral regions, the higher the spatial resolution, the smaller the specific eccentricity. (2) Crowding is seen as a key characteristic that distinguishes peripheral from foveal vision [20] (p. 36). It refers to the phenomenon that although an object is easily recognized alone, it cannot be recognized if it is closely surrounded by other objects (flankers). Crowding poses primarily a problem to peripheral vision because the critical distance between objects to avoid crowding increases rapidly with eccentricity. It can be described by a rule of thumb called “Bouma’s law” [29]; the critical distance between a target object and any surrounding object to avoid interference by crowding is half of the targets’ eccentricity. Thus, critical crowding distances are smallest in the near periphery, larger in the medium periphery, and largest in the far periphery. Furthermore, the outer parts of the far periphery (>70°) are especially sensitive to movement, in comparison to the remainder of the periphery: Contrast sensitivity is higher for moving objects than for non-moving objects. In the extreme periphery, near the border of the visual field, non-moving objects are not visible at all [19]. Despite these challenges for peripheral information processing, humans can perform complex tasks even in the far periphery of the visual field: For instance, participants were able to detect animals in large natural scenes, even at eccentricities of 70.5° [31]. In summary, inhomogeneity is an inherent property of the human visual system, especially in respect to physiological and performance differences between the central and the far peripheral visual field. It is therefore most plausible to assume that attentional processing across the visual field differs, too.

Critically, visual attention can be regarded as a compensation mechanism that allows the visual system to overcome the negative consequences of its inhomogeneity—low spatial resolution and crowding. Covert visual attention improves information processing for a relevant object by shifting visuo-spatial attention towards its location in the periphery (e.g., [32]; for a review, see [6]), while the center of gaze remains fixated elsewhere. For the near periphery, there is ample evidence that attention effects are influenced by the inhomogeneity of the visual field, at least quantitatively: attention effects change with retinal eccentricity in several experimental paradigms; they increase with eccentricity (1°–17°, e.g., [33]; ([34], Exp. 2); [35,36]). Furthermore, attention diminishes the influence of crowding [32]. Attention reduces the critical distance between objects above which no interference by crowding can be measured. On a neurophysiological level, visual attention is supposed to elicit these behavioral effects by enhancing spatial resolution via reducing receptive field size at attended locations and shifting receptive fields towards the focus of attention [6]. Thus, behavioral and neurophysiological evidence shows that—in the near periphery—inhomogeneity related-factors (e.g., eccentricity, crowding) are linked to visual attention in a bidirectional manner: On one hand, attention is influenced by these factors, but on the other, attention itself also influences inhomogeneity-related factors like crowding and spatial resolution.

A further, second reason why differences between near- and medium- or far-periphery attention might be likely is mixed empirical evidence from the two studies that already investigated attention in the medium or far periphery: Casteau and Smith [16], Exp. 1 & 3 demonstrated a qualitative difference between near (10°–20°) and medium eccentricities (30°–44°). They found a facilitative attention effect at near peripheral eccentricities, but failed to reveal such an effect for medium peripheral eccentricities. Their experiments used a detection task with placeholders and attention direction by a peripheral non-predictive cue. In contrast, Poggel et al. [17] failed to reveal a qualitative difference between different parts of the periphery (20°, 30°, 40°, 60°). In a near-threshold discrimination task without placeholders and a 100% predictive peripheral cue, they [17] found facilitative attention effects for all eccentricities. These conflicting results may be related to the following differences in the experimental paradigms that influence cueing effects in the near periphery: (1) task type (detection vs. discrimination), (2) cue predictiveness (non-predictive of target location vs. 100% predictive), and (3) placeholders (presence vs. absence). (1) Under certain conditions, attention effects for discrimination tasks are much larger than for detection tasks. Furthermore, with a detection task and non-predictive peripheral cues, facilitating attention effects are generally not easily obtained [37] (p. 41). Due to the inhomogeneity of the human visual field, the difference between discrimination and detection tasks might be even more pronounced at larger eccentricities. (2) Poggel et al. [17], using peripheral predictive cues, revealed cueing effects in medium and far periphery with peripheral predictive cues, while Casteau and Smith’s study ([16], Exp. 1 & Exp. 3), using non-predictive peripheral cues (exogenous attention), found no cueing effects in the medium periphery. Interestingly, Casteau and Smith [16] (Exp. 2. & Exp 3.) revealed cueing effects in the medium periphery with predictive central symbolic cues (endogenous attention). Thus, the cue’s predictiveness (peripheral or central) might be crucial for revealing cueing effects in the medium and especially in the far periphery beyond the EOMR. This would imply that exogenous attention alone, without an endogenous predictive cue, is not sufficient for eliciting cueing effects beyond the near periphery. (3) Using placeholders in combination with peripheral cues usually leads to larger and longer-lasting cueing effects than peripheral cueing without placeholders [37,38]. This positive placeholder effect is probably related to reduced spatial uncertainty and specific object-related attentional processes. However, if this positive placeholder effect is not reversed in medium and far periphery, it cannot explain the difference between the two studies [16,17] because it counteracts the observed result pattern. 

The present study investigates attention in a wider range of the visual field by expanding a classical paradigm of visual attention research to the medium and far periphery: The spatial cueing paradigm or “Posner cueing paradigm” (e.g., [1,11]) by Michael Posner. In the spatial cueing paradigm, attention is directed towards a specific location either by a peripheral cue (e.g., abrupt onset) or a central, symbolic cue (e.g., an arrow or a number). After a short interval, a target stimulus appears either at the cued location (valid trial) or at another non-cued location, often opposite to the cued location (invalid trial). In neutral trials, a target will always appear at a previously non-cued location because either no location or a location that could not be a target location was cued (e.g., fixation, whole display). Participants’ task is usually either to detect as fast and accurately as possible whether the target is present or absent (detection task, e.g., [11,16]) or to decide which of several targets is presented (discrimination task, e.g., [34]). The standard finding in the spatial cueing paradigm depends critically on cueing interval length (cue-target): For short cueing intervals (≤150–200 ms), reaction times (RT) are faster for validly than for invalidly cued trials; RTs of neutral trials usually lie in between. Including neutral trials allows calculating the benefits and costs of cueing separately: cueing benefits (neutral-valid) and cueing costs (invalid-neutral). However, calculating benefits and costs separately also provides specific problems [39] because a neutral cue might lead to changes in decision criteria as well as attentional distribution changes across the visual field. Therefore, it could be better to analyze cost + benefits together. Note that cost (invalid - neutral) + benefit (neutral - valid) equals the calculation of the cueing effect (invalid - valid). Effects on accuracy usually mirror the RT results: Participants are more accurate in validly than invalidly cued trials. However, for longer cueing intervals (>300 ms), the cueing effect reverses: RTs for valid trials are now slower than RTs for invalid trials. Theoretically, this reversal has often been interpreted as inhibition of a previously attended location (inhibition of return, IOR, [40,41]). However, a study by Danziger and Kingstone [42] implies that IOR is not necessarily dependent on exogenous attention but could also be a cue-related inhibition phenomenon with a considerably earlier onset (~50 ms) that is usually masked by facilitation at the attended location. Interestingly, IOR effects elicited by peripheral non-predictive cues vary strongly across the visual field depending on eccentricity [10,13,14,43]. Their size increases considerably beyond 15°–20°. Furthermore, the time course of IOR seems to change because IOR can be found for smaller cueing intervals (<300 ms). Bao et al. [14] found IOR effects with a single cueing procedure and a very small cueing interval of 150 ms for large eccentricities (20°, 25°, 30°), but not for smaller eccentricities (5°, 10°, 15°). Thus, at even larger eccentricities, cueing effects might not be found with cueing SOAs usually used in the near periphery due to an even earlier onset of IOR.

To give possible exogeneous attention effects in the medium and far periphery the best chance to be revealed, the present study uses: (1) a peripheral spatial cue that is not predictive of the succeeding target location, but will occur at each possible target location with equal probability. (2) It combines this peripheral non-predictive cue with a discrimination task and placeholders (“Was E or Mirror-E presented?”; see [44]) because this will probably lead to larger and more stable cueing effects than a detection task without placeholders. Furthermore, stimuli are presented at a wide range of target eccentricities, which means at one specific location in the near, medium, and far periphery (15°, 30°, 56° Exp. 1) and (15°, 35°, 56° Exp. 2). Distances between targets are chosen to minimize crowding because it could impair exogenous attention effects by reducing the peripheral cue’s effectiveness. Furthermore, trials with different eccentricities were presented randomly, not blocked to ensure better comparability with other studies using large eccentricities (e.g., [13,14,16]). Additionally, eye movements were controlled in Experiment 2 for two reasons. First, many previous studies on visual attention, e.g., [15], do not control for eye movements or deviations from a fixation area because it is assumed that the cueing SOAs in these studies are too short for the occurrence of eye movements (<200–150 ms). However, occasionally occurring faster eye movements would affect the retinal position of stimuli in paradigms without eye movement control and might also enhance or reduce the size of cueing effects. It is therefore important to know if results are affected by lack of eye movement control. Second, excluding and repeating trials in which the gaze deviated from the fixation area during the critical trial period also reduces intraindividual noise caused by various factors (e.g., fatigue, lapses in concentration) if they result in fixation errors. Thus, excluding not only trials with eye movements but also with fixation errors should further enhance the reliability of the measurement. 

The following specific hypotheses were tested: *Hypothesis (1)*. A facilitative cueing effect (valid RTs < invalid RTs) is expected at the near periphery eccentricity (15°). This hypothesis is derived from the vast number of near periphery visual attention studies showing facilitative cueing effects with similar experimental designs. *Hypothesis (2)*. Due to restricted empirical knowledge on visual attention beyond the near periphery, two alternative predictions are made for the medium and far periphery: *Hypothesis (2a).* Either, cueing effects rise with eccentricity (cueing near periphery < cueing medium periphery < cueing far periphery. This would constitute a quantitative difference between near, medium, and far periphery and is supported by rising cueing effects found in the near and medium periphery (e.g., [15,34,36]). Or *Hypothesis (2b)* cueing effects can only be found at the near (15°) and medium periphery (30° Exp. 1 or 35° Exp. 2) because 56° lies well beyond the EOMR ~45° and several studies using peripheral cues in combination with a detection task failed to reveal cueing effects beyond the EOMR [16,45,46,47]. This would constitute a qualitative difference between near and medium and the far periphery. *Hypothesis (3).* Furthermore, if cueing effects are revealed for all periphery ranges, a positive correlation between the different cueing effects might be expected: A participant with a large cueing effect at one eccentricity should have large cueing effects at the other eccentricities and vice versa. A positive correlation would provide strong support for the assumption that the same attentional process is at work in different parts of the periphery. However, less emphasis lies on Hypothesis 3 because analyzing cognitive experimental psychology tasks such as Posner Cueing with an individual difference approach has inherent problems ([48,49]). Usually, well-established experimental tasks that provide robust and replicable results like Posner cueing have a low between-subject variability. But a low between-subject variability has negative effects on the reliability of individual differences by impairing correlations with other factors [48]. Therefore, an individual difference approach usually requires much higher participant and trial repetition numbers to receive stable estimates of individual differences.

**Experiment 1**

## 2. Materials and Methods 

### 2.1. Participants

Twenty participants that were recruited at Bielefeld University (15 female, 5 male, 0 not indicated), median age 24 years (range 19–28 years), took part in Experiment 1. Before the experiment, they provided informed written consent. Participants received a compensation of 8 Euros per hour and had normal or corrected-to-normal visual acuity. The study was conducted in accordance with the Declaration of Helsinki and was approved by the Ethics-Committee of Bielefeld University (EUB-2015-094), approval date 21/07/2015.

### 2.2. Apparatus and Stimuli

The experiments were conducted in a dark room. Stimulus presentation was controlled by a Dell computer using Ubuntu Linux (14.04 LTS) and presented by a Propixx Projector (Full Version, Vpixx Technologies Inc., Saint-Bruno, QC, Canada) on a screen with front projection, with the projector mounted on a frame below the room ceiling that would allow to shift the distance between projector and screen between experiments. The projected image had a size of 2.08 m width × 1.17 m height and a diagonal value of 2.38 m. The projected image resolution was set to 1920 × 1080 pixels. Participants sat at a distance of 88 cm to the screen, their head fixated by a combined chin and head rest. The refresh rate of the projector was set to 120 Hz. Stimuli were a black fixation cross (1.4° × 1.4°,0.6 cd/m²), seven black rectangular placeholders (8.5° × 8.5°). The cue was an abrupt broadening of one of the placeholders from 4 px to 12 px. The target stimuli were the black letter E or a black Mirror-E (4.3° × 1.8°). Stimuli were presented on a medium gray background (23.6 cd/m²) only in a part of the projected image in a horizontal band of 1920 px × 360 px in the lower part of the screen. The rest of the projected image was set to black RGB (0.6 cd/m²). Participants’ responses were recorded by a ResponsePixx Button-Box with five buttons (Vpixx Technologies, Canada) arranged in the shape of a cross. The experiment was programmed using Matlab (2015b, The MathWorks Inc., Natick, MA, USA).

### 2.3. Procedure

**Figure 1:**
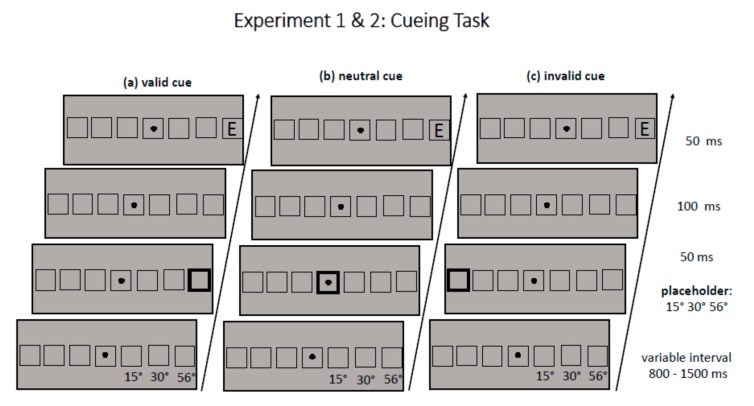
The figure shows the sequence of (**a**) validly, (**b**) neutrally, and (**c**) invalidly cued trials for Experiments 1 & 2, with the exception that the medium placeholder was presented at 35° in Experiment 2. Note that the distance between the placeholders is not drawn to scale.

Each trial started with the presentation of the fixation cross and seven square-like placeholders in the middle of the lower horizontal band of the screen. The middle placeholder surrounded the fixation cross, the other six placeholders were displayed in pairs of three to the left and three to the right of fixation with a horizontal distance (center-to-center) of 15°, 30.2° (medium periphery), and 56.2° (far periphery) from fixation (see Figure 1). (Note that eccentricity values reported in Section 2 are rounded to the first decimal place, whereas for better readability, eccentricity values are rounded to integer in all other parts of the manuscript.) After a variable interval (800–1200 ms), one of the seven placeholders was cued for 50 ms by broadening its frame from a width of 4 to 12 pixels. After an interstimulus interval of 100 ms and thus a cueing SOA of 150 ms, a target appeared for 50 ms either at the cued position (valid trial) or at the position opposite to the cued position (invalid trial). The target never appeared in the middle placeholder with the fixation cross. If the middle placeholder was cued, this was a neutrally cued trial (neutral trial). The task of participants in a given trial was to indicate as fast as possible, but also with high accuracy, whether the target letter (size 4.3°) was an E or Mirror-E by a press of the upper (yellow) and lower (blue) vertically aligned buttons on a ResponsePixx Button Box (Vpixx Technologies Inc., Saint-Bruno, QC, Canada). The mapping of the buttons was changed across participants. Participants responded with their right and left index fingers. Participants were instructed to look at the fixation cross during the whole trial. Participants with an even number received the mapping in which a yellow button press indicated response E and a blue button press indicated response Mirror-E. Participants with an odd number received the reversed mapping. Dependent variables were the choice reaction times of the participants and their error rate. Choice reaction times were computed from target onset. In Experiment 2, eye movements were recorded with an Eyelink 1000 Plus Desktop Mount (SR Research, Ottawa, ON, Canada) as fixation control, whereas no eye movements were recorded in Experiment 1. Overall, participants completed 720 trials, one-third each with a neutral cue, a valid cue, and an invalid cue. In each trial, one eccentricity level was cued (near, medium, far); all cueing conditions occurred equally often at both target sides (left, right). In half of the trials, target identity was *E* (360), in the other half *Mirror-E* (360). Target identity was equally distributed over all cueing conditions (120 trials per condition and target identity) and also over all eccentricities (120 trials per condition and target identity). However, due to a programming error, target identity was not equally distributed at each target side:

Ecc_15Left_(*valid*-E-40,ME-0; *neutral*-E-0,ME-40; *invalid*-E-40,ME-0);
Ecc_30Left_(*valid*-E-0,ME-40;*neutral*-E-40,ME-0;*invalid*-E-0,ME-40);
Ecc_56Left_(*valid*-E-40,ME-0;*neutral*-E-0,ME-40;*invalid*-E-40,ME-0);
Ecc_15Right_(*valid*-E-0,ME-40;*neutral*-E-40,ME-0;*invalid*-E-0,ME-40);
Ecc_30Right_(*valid*-E-40,ME-0;*neutral*-E-0,ME-40;*invalid-*E-40,ME-0);
Ecc_56Right_(*valid-*E-0,ME-40;*neutral-*E-40, ME-0;*invalid-*E-0,ME-40).

But note that neither target identity (E/ME) nor target side (left/right) are theoretically relevant for the present study. Nevertheless, RT results separated by target side can be seen in Supplementary Materials (Tables S1 and S2). All types of trials were presented randomly intermixed.

### 2.4. Results

For data analysis, median reaction times of correct responses were calculated for each participant and each condition of the within-subject factors Cueing (valid, neutral, invalid) and Eccentricity (15°, 30°, 56°). To exclude outliers, reaction times that were below or above three standard deviations (SD) of the mean were excluded from data analysis for each participant. For Experiment 1, a mean of (1.7%) percent of trials were excluded (range of excluded trials 2.5% to 0.27%). Note that for comparability with other studies, analyses were also calculated for median reaction times without outlier exclusion and mean reaction times with outlier exclusion (+/−3 SD). However, the result pattern remained the same. In Experiment 1, cueing effects for mean reaction times with outlier exclusion were Ecc 15° = 49 ms, Ecc 30° = 16 ms, and Ecc 56° = 80 ms; and cueing effects for median reaction without outlier exclusion were Ecc 15° = 48 ms, Ecc 30° = 18 ms, and Ecc 56° = 80 ms. Repeated measures ANOVAs with post-hoc pairwise t-tests were conducted for effects of factors Cueing and Eccentricity. If violations of sphericity occurred, degrees of freedom were corrected using Greenhouse-Geisser-Epsilon. If the assumption of normal distribution was violated, a non-parametric Friedman test was performed but only reported if the results differed from the ANOVA. Post-hoc Bonferroni-Holm corrected Wilcoxen tests were also only reported if they differed from the pairwise t-tests.

**Figure 2:**
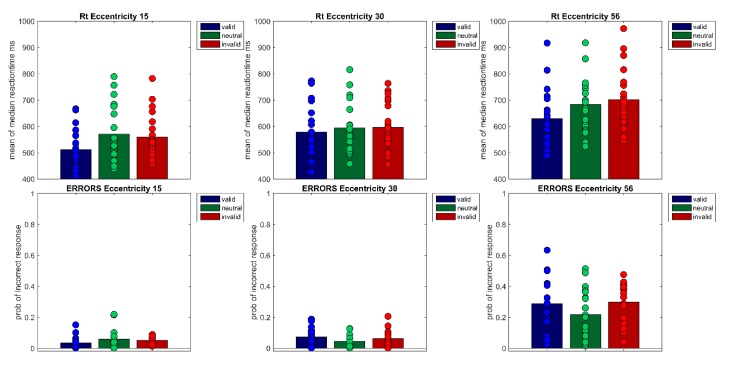
The figure shows the correct reaction times of Experiment 1 per Eccentricity (15°, 30°, 56°) × Cueing (valid, neutral, invalid). Bars show the mean of median reaction times across participants. Filled circles show the medians of each individual participant (upper row). The lower row shows the probabilities of incorrect responses for each of the nine conditions. Bars denote the mean probability across participants, filled circles the probabilities of each participant.

**Figure 3:**
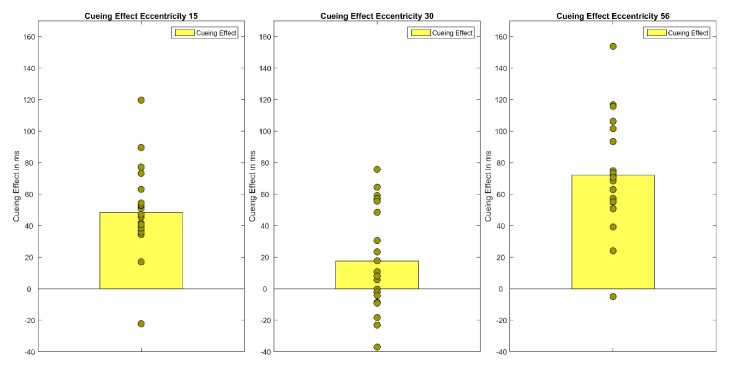
The figure shows the cueing effects (invalid RT-valid RT) for each eccentricity (15°, 30°, 56°). Bars show the mean of cueing effects across participants. Filled circles show the cueing effects of each individual participant.

A repeated measures ANOVA of correct median reaction times showed a main effect of Cueing, *F*(1.48,28.21) = 34.86, *p* < 0.001, *η*²*_G_* = 0.05 and Eccentricity, *F*(1.16,22.08) = 104.33 *p* < 0.001, *η²_G_* = 0.22 as well as a significant interaction (Cueing × Eccentricity), *F*(4,76) = 5.40, *p* < 0.001, *η²_G_* = 0.01 Bonferroni-corrected pairwise t-tests showed that mean reaction times rise with eccentricity (*M*_Ecc15_ = 547 ms, *SD* = 92; *M*_Ecc30_ = 590 ms, *SD* = 93; *M*_Ecc56_ = 671 ms, *SD* = 113, all *t’*s(59) < −7.33, all *p’*s < 0.001). Furthermore, valid and invalid RTs differed significantly, in the expected direction (*M_valid_* = 573 ms, *SD* = 105 vs. *M_invalid_* = 619 ms, *SD* = 114, *t*(59) = 9.11, *p* < 0.001). However, the neutral condition (*M_neutra_*_l_ = 616 ms, *SD* = 113) and the invalid condition (*M_invalid_* = 619 ms, *SD* = 114) did not differ from one another. Cueing effects (valid < invalid) were only found for the smallest eccentricity (*M_Ecc15 valid_* = 512 ms, *SD* = 74 vs. *M_Ecc15 invalid_* = 560 ms, *SD* = 86, *t*(19) = −7.40, *p* < 0.001) and the largest eccentricity (*t*(19) = −9.08, *p* < 0.001, *M_Ecc56 valid_* = 629 ms, *SD* = 109 vs. *M_Ecc56 invalid_* = 701 ms, *SD* = 115; see Figure 2 and Figure 3, Table 1); the cueing effect for the medium eccentricity was only marginally significant (*M_Ecc30 valid_* = 579 ms, *SD* = 96 vs. *M_Ecc30 invalid_* = 596 ms, *SD* = 91, *t*(19) = −2.42, *p* = 0.08).

A repeated measures ANOVA of arcsine transformed probabilities of correct responses revealed a main effect of Cueing, *F*(2,38) = 7.25, *p* <0.01, η²_G_ = 0.03, a main effect of Eccentricity, *F*(1.35,25.57) = 94.21, *p* < 0.001, *η*²*_G_* = 0.53, as well as a significant interaction Cueing × Eccentricity, *F*(2.38,45.24) = 3.16, *p* < 0.05, *η²_G_* = 0.04 Bonferroni-corrected post-hoc pairwise t-tests showed a difference between the invalid and the neutral condition, responses were slightly more accurate in the neutral condition than in the invalid condition (*M_neutral_* = 0.89, *SD* = 0.13 vs. *M_invalid_* = 0.86, *SD* = 0.14, *t*(59) = 2.91, *p* < 0.05). Note that mean values and SD are shown as untransformed values for better comprehensibility. The far eccentricity differed significantly from both other eccentricities (Ecc 15° vs. Ecc 56°, *t(*59) = 12.77, *p* < 0.001) and (Ecc 30° vs. Ecc 56°, *t*(59) = 12.79, *p* < 0.001. Responses were less accurate for the far eccentricity (*M_Ecc15_* = 0.95, *SD* = 0.04; *M_Ecc30_* = 0.94, *SD* = 0.05; *M_Ecc56_* = 0.73, *SD* = 0.15). Furthermore, t-tests showed a significant difference at eccentricity 15° between the valid and the invalid condition (*M*_ValidEcc15_ = 0.97; *SD* = 0.04; *M*_InvalidEcc15_ = 0.95; *SD* = 0.03; *t(*19) = 2.54, *p* < 0.05) and a significant difference between the valid and the neutral condition at eccentricity 30°, (*M*_ValidEcc30_ = 0.93; *SD* = 0.06; *M*_NeutralEcc30_ = 0.96; *SD* = 0.04; *t(*19) = −2.86, *p* < 0.01).

**Figure 4:**
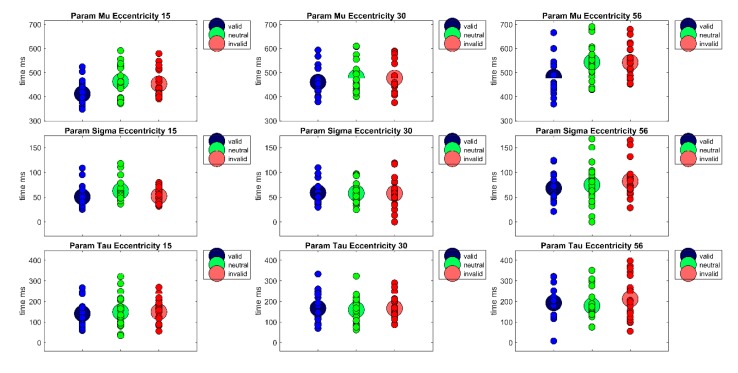
The figure shows the parameters of the ex-Gauss-fit (μ, σ, τ) for each Eccentricity (15°, 30°, 56°) × Cueing (valid, neutral, invalid) condition. Large circles show the condition mean and small circles show the values of each individual participant.

Furthermore, to separate perceptive and sensory processes from decision processes with respect to their contribution to reaction times, the reaction time distributions of each participant were fitted with an ex-Gauss function with 3 parameters (μ, σ, and τ) using the Matlab Toolbox by Lacouture and Cousineau [50]. Parameter μ denotes the measure for the central tendency of the Gaussian part of the function, parameter σ is a measure of the standard deviation of the Gaussian part of the function, and parameter τ is the measure of the exponential part of the function. Whereas parameter μ is often interpreted to reflect sensory or perceptual processes, parameter τ is frequently interpreted as a measure of the quality of decision processes or a measure for lapses in attention. A repeated measures ANOVA of parameter μ revealed a main effect of Cueing, *F*(2,38) = 24.93, *p* < 0.001, *η²_G_* = 0.09, a main effect of Eccentricity, *F*(1.35,25.56) = 67.14, *p* < 0.001, *η²_G_* = 0.21, as well as a significant interaction Cueing × Eccentricity, *F*(4,76) = 3.72, *p* < 0.01, *η²_G_* = 0.02 (see Figure 4). Bonferroni-corrected pairwise post-hoc t-tests revealed significant differences between the valid and the invalid condition (*M_valid_* = 451 ms, *SD* = 66 vs. *M_invalid_* = 490 ms, *SD* = 75, *t*(59) = −7.40, *p* < 0.001) and a significant difference between the valid condition and the neutral condition (*M_neutral_* = 495 ms, *SD* = 77, *t*(59) = −6.27, *p* < 0.001). This reflects a cueing effect in the expected direction (valid < invalid) of 39 ms. The main effect of Eccentricity is due to significant differences between all three eccentricity conditions (*M_Ecc15_* = 442 ms, *SD* = 61; *M_Ecc30_*= 472 ms, *SD* = 64; *M_Ecc56_* = 522 ms, *SD* = 78, all *t’*s(59) < −6.07, all *p’*s < 0.001); reflecting a rise in reaction times with eccentricity. At the eccentricity level, there was a cueing effect of 42 ms at eccentricity of 15° (*M_Ecc15 Valid_* = 411 ms, *SD* = 48 vs. *M_Ecc15 Invalid_* = 453 ms, *SD* = 56, *t*(19) = −6.40, *p* < 0.001), and a significant difference between the neutral and the valid condition (*M_Ecc15 Neutral_* = 463 ms, *SD* = 67, *t*(19) = −4.22,*p* < 0.01). At eccentricity of 56°, there was a cueing effect of 61 ms (M_Ecc56 Valid_ = 481 ms, *SD* = 72 vs. *M_Ecc56 Invalid_* = 542 ms, *SD* = 70, *t*(19) = −5.39, *p* < 0.001) and a significant difference between the valid and the neutral condition at this eccentricity (*M_Ecc56 Neutral_* = 543 ms, *SD* = 79, *t*(19) = −4.28, *p* < 0.01). At eccentricity 30°, the cueing effect of 17 ms is only marginally significant (*M_Ecc30valid_* = 460 ms, SD = 59 vs. *M_Ecc30invalid_* = 477 ms, *SD* = 70, *t*(19) = −2.41, *p* = 0.08). However, the Bonferrroni-Holm corrected Wilcoxon signed rank test for the cueing effect at eccentricity 30° was not significant, *p* = 0.91.

A repeated measures ANOVA of parameter σ revealed only a main effect of Eccentricity, *F*(1.51,28.78) = 11.91, *p* < 0.001, *η²_G_* = 0.10. Bonferroni-corrected post-hoc pairwise t-tests revealed a significant difference between the near and far eccentricity (*M_Ecc15_* = 55 ms, *SD* = 20 vs. *M_Ecc56_* = 75 ms, SD = 35, *t*(59) = −4.21, *p* < 0.001) and the medium and far eccentricity (*M_Ecc30_* = 58 ms, *SD* = 24 vs. *M_Ecc56_* = 75 ms, *SD* = 35, *t*(59) = −3.49, *p* < 0.01). Thus, reaction times vary more strongly at the largest eccentricity.

A repeated measures ANOVA of parameter τ revealed only a main effect of Eccentricity, *F*(2,38) = 17.97, *p* < 0.001, η²_G_ = 0.07. This main effect is due to significant differences between all eccentricities, (*M*_Ecc15_ = 146 ms, *SD* = 65; *M*_Ecc30_ = 164 ms, *SD* = 66; *M*_Ecc56_ = 194 ms, *SD* = 85, all *t*’*s*(59) < −2.99, all *p’s* < 0.05). Thus, attentional lapses or other decision processes seem to rise with eccentricity.

Furthermore, individual differences in cueing effect size (cost + benefit) are analyzed by correlating cueing effects between the three eccentricities. None of the correlations was significant (near and far eccentricity, *r*(18) = 0.18, *p* = 0.45, medium and far eccentricity, *r*(18) = 0.17, *p* = 0.46, near and medium eccentricity, *r*(18) = −0.16, *p* = 0.50).

### 2.5. Discussion

Experiment 1 demonstrates a cueing effect by peripheral cues of 48 ms at the smallest eccentricity (15°) and 72 ms at the largest (56°), but only a marginally significant cueing effect of 17 ms at the medium eccentricity (30°). As predicted by Hypothesis 1*,* a cueing effect was revealed at the near periphery of 15°. In accordance with the quantitative-difference Hypothesis 2a, this cueing effect rises to 72 ms at the far periphery eccentricity. Finally, in contradiction to Hypothesis 3, Experiment 1 could not reveal any positive correlation between the size of cueing effects at the different eccentricities. However, due to the study’s experimental design (e.g., number of repetitions per condition, number of participants), which is not ideal for individual difference analysis, individual cueing effects at each eccentricity probably do not provide stable estimates of individual cueing effects. Thus, the lack of positive correlations between cueing effect size at different eccentricities should not be interpreted as evidence for different attentional mechanisms in near, medium, and far periphery. 

On a theoretical level, Experiment 1 implies that the EOMR—eccentricity range that can be reached by an eye movement alone—is not necessarily a boundary for exogeneous attention because the 72 ms cueing effect at 56° lies well beyond the EOMR of ~45° in the far periphery. By contrast, due to the close link between visual attention and eye movements, Smith and colleagues assume that cueing effects cannot be elicited by non-predictive peripheral cues outside the EOMR. This assumption is derived from premotor-theory or hypothesis of attention [51], which assumes that the activation of the eye movement system is necessary for eliciting attention shifts. The premotor theory assumes that covert attention shifts are in fact eye movements that have been prepared, but not executed. Since eye movements can neither be executed nor prepared towards a location beyond the EOMR, a strong version of the premotor hypothesis implies that *exogenous* as well as *endogenous* attention cannot be deployed outside the EOMR. However, based on their empirical findings, Smith and colleagues proposed a weaker version of the premotor hypothesis in which only exogeneous attention (peripheral non-predictive cues) fails to elicit cueing effects outside the EOMR, but endogenous attention (central symbolic predictive cues) does elicit cueing effects also beyond the EOMR. They argued that the eye movement system is not necessarily involved in endogenous attention because the participants’ knowledge about the cue’s predictiveness biases the visual system towards the cued location by top-down cognitive processes and expectations without involvement of the oculomotor system. Nevertheless, the EOMR could provide an explanation for the reduced cueing effect at 30° eccentricity, if individual levels of the EOMR for many participants lay well below the mean EMOR of ~45°. Thus, no cueing effects would be found for these participants at the medium eccentricity because it would be already located beyond their individual EOMR range. Indeed, Casteau and Smith [16] measured much smaller eccentricities (30°–44°) as individual EOMR limits. However, this explanation fails to account for the cueing effect found in the far periphery. If lower individual EOMR limits are responsible for the reduced cueing effect at the medium periphery, certainly no cueing effect should be found beyond the EOMR in the far periphery. It is therefore more likely that other factors caused the reduced and only marginally significant cueing effect at eccentricity 30°. These other factors, which will be discussed below, might also provide an alternative explanation for the results of Casteau and Smith [16], since they did not test eccentricities in the far periphery. 

Basically, the reduced and marginally significant cueing effect at eccentricity 30° could be explained either due to inhomogeneity-related phenomena (crowding, cue effectiveness, eye movements, IOR) or methodological factors (e.g., low power, low measurement reliability). The first inhomogeneity related factor that might have contributed to the reduced cueing effect at the medium eccentricity of 30° is crowding. Crowding should impair the medium position most strongly because it is located between two placeholders at each side. However, the far position has only one placeholder to its side. Even though the near position is also located between two placeholders (fixation and medium placeholder), it probably experiences less crowding from the fixation placeholder. Furthermore, although strong crowding effects are unlikely with the chosen eccentricities, crowding cannot be ruled out completely. “Bouma’s rule” adapted by Strasburger et al. [20] would require a center-to-center distance of half the eccentricity plus the stimulus width, which could not be realized due to restrictions of the experimental setup. However, in this case, crowding should impair discrimination accuracy at the medium location, which was not the case (see Figure 2). Moreover, cueing usually has a diminishing effect on crowding [32]. It reduces the critical distance between two stimuli above which no interference by crowding occurs. The only way in which stronger crowding at the medium placeholder could be responsible for the reduced cueing effect is by reducing the effectiveness of the peripheral cue itself. The medium cue’s effectiveness could be especially impaired because in contrast to the far cue, it is surrounded by two placeholders instead of one, and in contrast to the near cue, these two surrounding placeholders both have low spatial resolutions. Thus, via reducing peripheral cue effectiveness, crowding might explain the reduced cueing effect at the medium eccentricity. Additionally, the medium cue’s effectiveness could also have been reduced due to other factors than crowding. For instance, the medium 30°eccentricity chosen in Experiment 1 lies at the outer border of another performance plateau in the visual field, that of light sensitivity [20,52,53].

The third inhomogeneity-related factor that could explain the reduced cueing effect is eye movements in the direction of the peripheral cue. However, this does not seem very likely since eye movements in the direction of the cue should, if anything, rather increase reaction time differences between the valid and invalid condition, but probably not diminish it. A fourth inhomogeneity-related explanation might be provided by an IOR-related functional subdivision of the visual field beyond 15°, the smallest eccentricity used in Experiment 1. In a detection task Bao et al. [14] found a faster time course of IOR at 20°, 25° and 30° because IOR could be elicited with a cueing SOA of 150 ms. Crucially, this cueing SOA was used in the present study and is usually far too short to elicit IOR in the near periphery. IOR shows a slower time course in the near periphery, it is usually not detected with cueing SOAs shorter than 300 ms. Thus, the reduced cueing effect might be due to interindividual differences in IOR time course at larger eccentricities, thus reducing the overall cueing effect at eccentricity of 30°. However, if this is the case, the onset of IOR at eccentricity 56° must be considerably later to explain the large overall cueing effect found at this eccentricity.

However, the reduced and only marginally significant cueing effect at 30° eccentricity could also be explained by methodological factors. Reduced power, reliability or effects of the specific sample might have led to a random finding of a non-existent or reduced cueing effect at the medium periphery and might not bear theoretical implications at all. This explanation would be excluded by replicating this finding in the medium periphery.

Furthermore, Experiment 1 showed rising reaction times with eccentricity. This is in accordance with previous studies that demonstrated an increasing effect of eccentricity on reaction times for stimuli not scaled for cortical magnification, for a review see [20] (p. 17), [54,55]. Interestingly, the increase in reaction times in Experiment 1 with an increase about 3 ms per degree of visual angle is overall somewhat larger than the increases described by Strasburger et al. [20], with a mean size of about 2 ms per degrees of visual angle. However, note that the stimuli used in the present study are larger and that stimulus size should have a strong effect on the increase in reaction times. These eccentricity effects on reaction times are probably due to factors that reflect the inhomogeneity of the visual system, as for instance cortical magnification, larger and fewer receptive fields at more eccentric locations that result in lower spatial resolution and stronger susceptibility to crowding.

Since empirical evidence on visual attention in the medium and far periphery is sparse, it is the aim of Experiment 2 to replicate the main results of Experiment 1—cueing effects in the near and far periphery, and a non-existent or a reduced cueing effect in the medium periphery—in a slightly changed experimental design. First, eye movements as well as other gaze deviations from the designated fixation area (fixation errors) are controlled for in Experiment 2 by discarding and repeating trials with fixation errors in the critical trial period. In addition to eye movements elicited by the peripheral cue, this fixation control will also eliminate unspecific eye movements and any other deviation from fixation. This should lead to a reduction of intra-individual noise and consequently an increase in reliability because it also eliminates trials in which participants did not comply with the fixation task instructions correctly due to various reasons (e.g., eye movements, fatigue, momentary lapses of concentration). Moreover, fast eye movements might have enhanced cueing effects at the near and far periphery in Experiment 1. Furthermore, a different medium eccentricity of 35° degrees is used in Experiment 2, because 30°–35° constitutes the outer border of another performance plateau in the visual field, that of light sensitivity [20,52,53]. If a reason for the reduced cueing effect at the medium eccentricity is a reduced effectiveness of the attentional cue due to its location near this border, a slight shift of the medium eccentricity might help to reveal a stronger cueing effect. But most importantly, replicating the non-existent cueing effect at a slightly shifted location would indicate a robust and real theoretical effect, because it is very unlikely that a theoretically relevant effect would be found only at the exact location of 30°.

**Experiment 2**

Experiment 2 is a replication of Experiment 1 with control of possible eye movements and a slightly changed medium target location of 35.2°. Eye movements were monitored with an Eyelink 1000 Plus Desktop Mount (SR Research, Ottawa, ON, Canada) monocularly at a refresh rate of 2000 Hz. In all participants, the left eye was tracked. For offline analysis, eye tracking data were downsampled to 250 Hz. Materials and Methods of Experiment 2 were very similar to Experiment 1; hence, only deviations from Materials and Methods with respect to Experiment 1 are reported here.

## 3. Materials and Methods

### 3.1. Participants

Seventeen participants recruited at Bielefeld University took part in Experiment 2 (7 male, 11 female, no participant gender not indicated, mean age 25 years, range 21 to 30 years). One participant’s session had to be aborted because she made too many fixation errors. The data of one further participant had to be excluded because the median of reaction times in several conditions was larger than 1000 ms.

### 3.2. Apparatus and Stimuli

In addition to apparatus and stimuli described in Experiment 1, Experiment 2 used an Eyelink 1000 Plus Desktop Mount (SR Research, Ottawa, ON, Canada) to monitor for possible eye movements.

### 3.3. Procedure

In addition to the procedure already described in Experiment 1, the position of the left eye was monitored at 2000 Hz. A trial was repeated if the participants did not fixate in a circular area of 2.5° around the fixation cross before the onset of the cue and after the offset of the target. If participants did not fixate correctly, the fixation cross became red after the trial. Furthermore, additional trials were excluded offline from the analysis, if eye positions outside an area of 2° around the fixation cross were registered in the critical trial interval between cue onset and target offset. For the offline analysis, data was downsampled to 250 Hz. On average, 6.6 percent of trials had to be repeated and 1.8 percent of trials were excluded offline because participants fixated outside the specified area. 

### 3.4. Results

**Figure 5:**
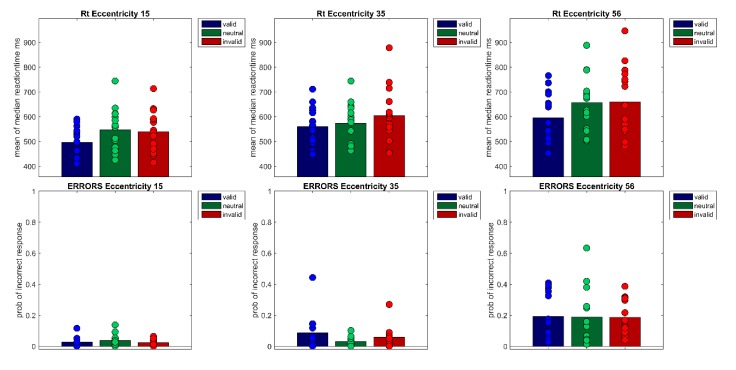
The figure shows the correct reaction times of Experiment 2 per Eccentricity (15°, 35°, 56°) × Cueing (valid, neutral, invalid). Bars show the mean of median reaction times across participants. Filled circles show the medians of each individual participant (upper row). The lower row shows the probabilities of incorrect responses for each of the nine conditions. Bars denote the mean probability across participants, filled circles the probabilities of each participant.

**Figure 6:**
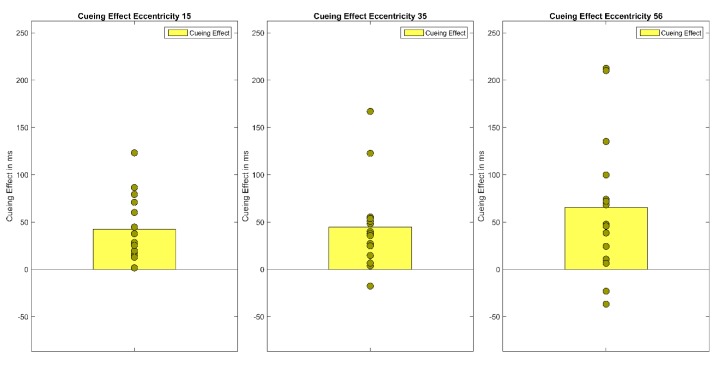
The figure shows Experiment 2′s cueing effects (invalid RT-valid RT) for each eccentricity (15°, 35°, 56°). Bars show the mean of cueing effects across participants. Filled circles show the cueing effects of each individual participant.

First, median reaction times of correct responses were calculated for each participant and each condition of the within-subjects factors Cueing (valid, neutral, invalid) and Eccentricity (15°, 35°, 56°). To exclude outliers, reaction times that were above or below 3 SD of the mean were excluded from data analysis. On average, 1.6 percent of trials were excluded as outliers. Note that as previously demonstrated in Experiment 1, analyses with median reaction times without outlier exclusion and mean reaction times with outlier exclusion lead to the same result pattern. A repeated measures ANOVA of correct median reaction times showed a main effect of Cueing *F*(1.40,19.55) = 21.03, *p* < 0.001, *η²_G_* = 0.05 and Eccentricity, *F*(1.30, 18.27) = 72.99, *p* < 0.001, *η²_G_* = 0.18 as well as a significant interaction (Cueing × Eccentricity), *F*(4, 56) = 2.99, *p* < 0.05, *η²_G_* = 0.01 (see Figure 5 and Figure 6). Bonferroni-corrected pairwise t-tests showed that the main effect of cueing is due to significant differences between the valid condition (*M* = 550 ms, *SD* = 91) and the invalid condition (*M* = 601 ms, *SD* = 123, *t*(44) = −6.33, *p* < 0.001), and thus a cueing effect of 51 ms, as well as a significant difference between the valid and the neutral condition (*M* = 592 ms, *SD* = 103, *t*(44) = −5.91, *p* < 0.001). The main effect of Eccentricity is due to reaction times rising with eccentricity; significant differences were found between all three eccentricity levels (*M*_Ecc15_ = 527 ms, *SD* =80; *M*_Ecc35_ = 579 ms, *SD* = 92; *M*_Ecc56_ = 637 ms, *SD* = 121), all *t’*s(44) < −6.75, all *p’*s < 0.001). Bonferroni-corrected pairwise t-tests at each eccentricity level showed cueing effects in the expected direction at all eccentricities (*M*_validEcc15_= 497 ms, *SD* = 62, *M_i_*_nvalidEcc15_ = 539 ms, *SD* = 83, *M*_validcEcc35_ = 559 ms, *SD* = 80, *M_i_*_nvalidEcc35_ = 604 ms, *SD* = 111, *M*_validEcc56_ = 595 ms, *SD* = 103, *M_i_*_nvalidEcc56_ = 660 ms, *SD* = 143, all *t’s*(14) < −3.43, all *p’s* < 0.05), but the neutral condition differed only significantly from the following conditions (*M*_neutralEcc15_ = 547 ms, *SD* = 87 vs. *M*_validEcc15_ = 497 ms, *SD* = 62, *M*_neutralEcc56_ = 656 ms, *SD* = 110 vs. *M*_validEcc56_ = 595 ms, *SD* = 103, all *t’s*(14) < −4.05, all *p’s* < 0.01). 

**Figure 7:**
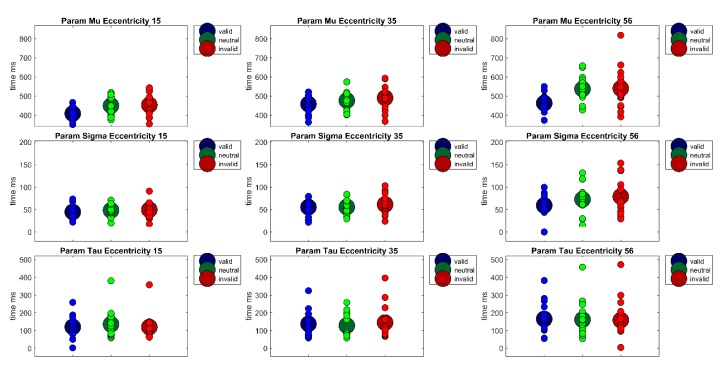
The figure shows the parameters of the ex-Gauss-fit (μ, σ, τ) for each Eccentricity (15°, 35°, 56°) × Cueing (valid, neutral, invalid) condition in Experiment 2. Large circles show the condition mean and small circles show the values of each individual participant.

A two-way ANOVA of arcsine-transformed percent correct data with factors Cueing × Eccentricity revealed only a main effect of Eccentricity, *F*(1.29,18.05) = 57.45, *p* < 0.001, *η²_G_* = 0.43. This main effect indicated decreasing accuracy with increasing eccentricities (*M*_Ecc15_ = 0.97, *SD* = 0.03, *M*_Ecc35_ = 0.94; *SD* = 0.08, *M*_Ecc56_ = 0.81, *SD* = 0.14, all *t’*s(44) > 2.89, all *p’*s < 0.05). Note that all post-hoc tests were performed on arcsine-transformed data, although untransformed means and standard deviations are reported here for better comprehensibility. As in Experiment 1, reaction time distributions were fitted with an ex-Gauss function using the Matlab Toolbox by [50]), see Figure 7. A repeated-measures ANOVA of parameter μ revealed a main effect of Cueing, *F*(2,28) = 17.88, *p* < 0.001, *η²_G_* = 0.12 as well as a main effect of Eccentricity, *F*(2,28) = 55.25, *p* < 0.001, *η²_G_* = 0.21 Bonferroni corrected pairwise t-tests revealed that parameter μ differed for the valid and the invalid condition (*M*_valid_ = 443 ms*, SD =* 51*, M*_invalid_ = 494 ms, *SD* = 86, *t*(44) = −4.79, *p* < 0.001) and the valid and the neutral condition (*M*_neutral_ = 487 ms, *SD* = 67, *t*(44) = −5.97, *p* < 0.001). Thus, parameter μ shows a cueing effect of 51 ms. The main effect of Eccentricity is due to rising reaction times with increasing eccentricity (*M*_Ecc15_ = 437 ms, *SD* = 52, *M*_Ecc35_ = 475 ms, *SD* = 55, *M*_Ecc56_ = 513 ms, SD = 87; all *t’*s(44) < −3.53, all *p’*s < 0.01). A repeated measures ANOVA of parameter σ revealed only a significant main effect of Eccentricity, *F*(2,28) = 22.25, *p* < 0.001, *η²_G_* = 0.14 Bonferroni-corrected pairwise t-tests revealed that the main effect is due to significant differences between all eccentricities (*M_Ecc15_* = 47 ms, *SD* =15; *M_Ecc35_* = 57 ms, *SD* =19; *M_Ecc56_*= 70 ms, *SD* =33; all *t’*s(44) < −2.60, all *p’*s < 0.05). This might be interpreted as an indicator that the perceptual part of reaction times is more variable at larger eccentricities. A repeated measures ANOVA of parameter τ revealed a main of Eccentricity, *F*(2,28) = 9.87, *p* < 0.001, *η²_G_* = 0.03 Bonferroni-corrected pairwise *t*-tests revealed that the main effect is due to a significant difference between the near and far eccentricity (*M_Ecc15_* = 125 ms, *SD* = 71 vs. *M_Ecc56_* = 162 ms, *SD* = 103, *t*(44) = −4.36, *p* < 0.001) as well as a significant difference between the medium and far eccentricity (M_Ecc35_ = 137 ms, *SD* = 77 vs. *M_Ecc56_* = 162 ms, *SD* = 103, *t*(44) = −2.71, *p* < 0.05). 

Furthermore, as in Experiment 1, individual differences in cueing effect size (cost+benefit) are analyzed by correlating cueing size between the three eccentricities. There was a moderate positive correlation between cueing size of the near and far eccentricity, *r*(13) = 0.62, *p* < 0.05 Neither the correlation between the medium and the far eccentricity*, r*(13) = 0.37, *p* = 0.18, nor the correlation between the near and medium eccentricity were significant, *r*(13) = 0.37, *p* = 0.17.

### 3.5. Discussion 

Overall, Experiment 2 replicated the results of Experiment 1 with additional eye position control and a slightly changed medium eccentricity position of 35°. Like Experiment 1, it revealed the expected cueing effect in the near periphery (Ecc 15° = 43) and a larger cueing effect in the far periphery (Ecc 56° = 66 ms). However, the non-existent or reduced cueing effect in the medium periphery was not replicated; instead, Experiment 2 revealed a substantial cueing effect (Ecc 35° = 45 ms). Replicating the main findings of Experiment 1 was especially important since, as already discussed broadly, few studies on spatial cueing have been conducted in medium and far periphery so far. As predicted in Hypothesis 2a, Experiment 2 shows at least numerically rising cueing effects (Ecc 15° = 43 ms, Ecc 35° = 45 ms, Ecc 56° = 66 ms) and no reduction of the cueing effect at the medium eccentricity as in Experiment 1. Furthermore, as predicted in Hypothesis 3, Experiment 2 revealed a positive moderate correlation *r* = 0.61 between the near and far eccentricity; lower correlations with the medium eccentricity were not significant. Thus, it can be concluded that the same attentional mechanism is at work in the near and far periphery. Furthermore, as expected, intra-individual noise reduction due to eye movement and fixation control in Experiment 2 helped to reveal this effect, because the near and far experimental condition differed from Experiment 1 only in this respect. However, whether the same attentional mechanism also operates in the medium periphery is less clear and should be investigated in future research with an experimental design more tuned to an inter-individual difference approach.

Thus, Experiment 2 is in complete accordance with the quantitative Hypothesis 2a that predicts rising cueing effects with rising eccentricity. However, does this indicate that the reduced cueing effect at the medium position in Experiment 1 is due to deviations from fixation and small eye movements that could not be detected due to a lack of eye tracking control or fixation control? This does not seem likely for undetected eye movements elicited by the peripheral cue because they should be drawn primarily in the direction of the peripheral cue, that means in the direction of the target in the valid condition but away from the target in the invalid condition. Thus, eye movements towards the peripheral cue should enhance cueing effects rather than diminish them. However, eye movements and deviations from the fixation area induced by various other factors (e.g., lapse of concentration, fatigue) might have randomly been incorporated in the medium position cueing effect in greater proportion, thereby reducing the cueing effect. The cueing effect of 45 ms might also be due to the shift of medium eccentricity from 30° to 35°. Reasons for this difference might be reduced cue effectiveness in Experiment 1, either due to crowding or to a location at the border of the often-found light sensitivity plateau or a different time course of IOR between the two locations. However, none of these options seems especially likely, since it was only a small shift of 5°. Thus, although Experiment 2 cannot finally answer the question of what caused the reduced cueing effect in the medium eccentricity, it indicates that the effect is not very robust or stable because it could not be replicated when the test location in the medium periphery was slightly shifted and eye movements and fixation errors are controlled for. It seems unlikely that a theoretically relevant effect could only be revealed at the specific location of 30°. Finally, as in Experiment 1, Experiment 2 shows rising reaction times with eccentricity, which is in accordance with previous findings, [20,54,55] and probably linked to the inherent inhomogeneity of the visual field. 

## 4. General Discussion

The present study explored visual attention beyond the near periphery to which most visual attention studies are restricted. More specifically, it investigated the question whether exogenous attention elicited by non-predictive peripheral cues shows qualitative or quantitative differences between near periphery attention and medium or far periphery attention. A qualitative difference would indicate either no cueing effect or a reversed inhibitory cueing effect (valid RTs > invalid RTs) in the medium or far periphery since facilitative cueing effects are expectable (valid RTs < invalid RTs) in the near periphery. A quantitative difference would indicate a difference in cueing effect size, but not in direction or even existence. 

Theoretically, a qualitative difference between near, medium, and far periphery might be predicted due to the visual field’s inhomogeneity because it leads to very different conditions under which attention must operate across the periphery. Furthermore, a qualitative difference between near/medium vs. far periphery is predicted by the premotor hypothesis of attention [51] and studies by Smith and colleagues [16,45,46,47]. Due to the close coupling between (visual) attention and eye movements e.g., [44], the premotor hypothesis assumes that activating the eye movement system is necessary for eliciting attention shifts. In its strong version, it predicts that neither exogeneous nor endogenous attention effects should be found beyond the EOMR, because covert attention shifts are conceived of as prepared, but unexecuted, eye movements. Since eye movements cannot be prepared or executed to locations beyond the EOMR, attention shifts towards these locations are impossible—provided attention shifts are indeed non-executed eye movements. In a weaker version of the premotor hypothesis Smith and colleagues proposed, based on their empirical findings, that only exogenous attention shifts are not possible beyond the EOMR. But in contrast to the premotor hypothesis of attention and the possible implications of the visual system’s inhomogeneity, the present study revealed overall no qualitative differences for the different parts of the periphery. Cueing effects were found from near periphery to far periphery for almost all investigated eccentricities, but with one critical exception: Experiment 1 revealed only a marginally significant cueing effect of 17 ms at eccentricity 30° in the medium periphery. Thus, as in the near periphery, attention seems to work as compensation mechanism for the visual field’s inhomogeneity in medium and far periphery. On a physiological level, this compensation mechanism would probably be linked to Area Prostriata ([22,23]), specialized on fast-moving stimuli in the far periphery, which implies an important role in visual orienting and exogenous attention, especially in the far periphery. 

However, Experiment 1 and 2 revealed small-to-medium *quantitative differences* between attention effects in different parts of the periphery: Somewhat larger cueing effects were found in the far periphery (Experiment 1: 72 ms vs. 48 ms, Experiment 2: 66 ms vs. 43 ms) than in the near periphery. What mechanisms might explain the rising size of cueing effects towards the far periphery? In consideration of the IOR increase beyond 15°–20°, Bao and colleagues [13,14] suggested that two different attention systems are at play in the periphery, differentiating the near periphery from the medium and far periphery. The second attentional system leads to a stronger modulation of exogenous attention effects than the first due to its more numerous projections to the superior colliculus in the medium periphery. However, the moderate positive correlation between cueing effects in the near and far periphery in Experiment 2 indicates that the same attentional mechanism is at play at both locations. This speaks clearly against the idea of attention in the near periphery being governed by a different attention system than that operating in the medium and far periphery. However, due to the probably not particularly stable estimates of individual cueing effects, no decision can be made with respect to the medium eccentricity. It might be a lack of experimental power or a different mechanism. Mapping out the region in medium periphery between 25° and 45° more closely and also measuring the time course of cueing effects provides an interesting line for future research. Furthermore, since exogenous attention enhances spatial resolution in the periphery via shifting receptive fields towards the attended location and shrinking of receptive fields at the attended location [6], it seems plausible that attention is most advantageous in the far periphery where receptive fields are especially large and sparse. Furthermore, in accordance with previous studies (for an overview, see [20], p. 17, [54,55]), reaction times increased with rising eccentricity in both experiments. Reaction time differences across eccentricity are probably linked to the inhomogeneity of the visual field, especially differences in receptive field size and spatial summation. 

In addition to the already discussed main findings of the present study, ex-Gauss fitted reaction times distributions support the reported findings but allow further inferences about the nature of these processes: Cueing effects for all eccentricities were revealed in the measure of the central tendency parameter μ, except an only marginally significant cueing effect at 56° in Experiment 2 (Experiment 1: Ecc_15_ = 42 ms*, Ecc_30_ = 17 ms*, Ecc_56_ = 61 ms*; Experiment 2: Ecc_15_ = 45 ms*, Ecc_35_ = 32 ms*, Ecc_56_ = 77 ms). Additionally, reaction time reliability was higher at the far periphery eccentricity, reflected in a larger parameter σ of the fitted reaction time distributions (Experiment 1: Ecc_15_ = 55 ms, Ecc_30_ = 58 ms, Ecc_30_ = 75 ms; Experiment 2: Ecc_15_ = 47 ms, Ecc_35_ = 57 ms, Ecc_56_ = 70 ms). Furthermore, in both experiments, decision processes or attentional lapses seemed to be more variable in the far periphery. Parameter τ was larger for the largest eccentricity (Experiment 1: Ecc_15_ = 146 ms, Ecc_30_ = 164 ms; Ecc_56_ = 194 ms, Experiment 2: Ecc_15_ = 125 ms, Ecc_35_ = 137 ms, Ecc_30_ = 162 ms). However, some caution in interpreting these parameters might be in order due to the ongoing debate surrounding the question whether specific cognitive processes can be mapped to the different parameters of the ex-Gauss function or not [56].

Finally, how are the present findings related to the few studies that previously investigated visual attention beyond the near periphery? The present study’s main findings are in accordance with the results of Poggel et al. [17], who showed cueing effects up to eccentricities of 60°. However, they are in disagreement with the results of Casteau and Smith [16], who found no cueing effects in a range of 30°–44°. They are also in indirect disagreement with previous eye-abduction paradigm studies by Smith and colleagues that failed to reveal cueing effects with peripheral cues beyond the EOMR, e.g., [41,42,43]. How can these contradicting findings be explained? Firstly, among other differences between the three studies that will be elaborated on in more detail below, Smith and colleagues used a detection task whereas the present study as well as Poggel et al. [17] used a discrimination task. This difference alone might be crucial since, at least in the near periphery; (facilitative) cueing effects by non-predictive peripheral cues with short cueing SOAs are usually substantially larger for discrimination tasks than for detection tasks (Chica et al. [37], see Table 1, p. 38). In their paper, the mean cueing effect over several experiments was 13 ms for the detection task experiments but more than twice as large for the discrimination task experiments (29 ms). This detection-discrimination task difference might be even more pronounced in the medium and far periphery because detection and discrimination should be affected differently by inhomogeneity-related factors like crowding and spatial resolution. Since crowding would affect discrimination tasks more strongly, attention might be especially beneficial for discrimination tasks, thus leading to larger differences between validly and invalidly cued targets. Furthermore, even in the near periphery, cueing effects by peripheral cues are more easily revealed with discrimination tasks. As stated by Chica et al. [37] in their informative review article: “Contrary to what it is usually assumed by most researchers, when using detection tasks and spatially nonpredictive peripheral cues, facilitation is not frequently observed even at short SOAs (see Table 1), unless there is a temporal overlap between the cue and target pp. 41–42”. It is also important to note that IOR is a robust finding in detection tasks and appears usually at shorter cueing SOAs for detection tasks than for discrimination tasks [37], a difference that might become even more pronounced due to the changed time course of IOR in the medium and far periphery found in detection tasks [14]. Furthermore, inter-individual differences in the onset of IOR, which could be earlier for detection tasks alone, might conceal cueing effects by peripheral cues in the study of Casteau and Smith [16]. Remarkably though, Casteau and Smith [16] did not find IOR effects even for eccentricities below the EOMR (10°–20°) and cueing SOAs that would usually lead to IOR (400 ms, 600 ms, Experiment 1 or 400 ms Experiment 3). However, if the time course of IOR in medium and far periphery, as indicated by the results of Bao and colleagues, has an earlier onset than in the near periphery, but shows also, as reaction times in general, a high inter-individual variability, cueing effects for peripheral cues might be concealed by early onset of IOR for some participants in the study by Casteau and Smith [16]. Thus, due to individual differences in IOR time course, cueing effects beyond the EOMR and IOR might average out one another for peripheral cues in the study of Casteau and Smith, but probably not for central cues. This seems especially likely since “IOR is exclusively behaviorally observed with spatially non-predictive peripheral cues”, [37] (p. 40)”. Thus, it should not affect the endogenous cueing effects in the study by Casteau and Smith [16].

To summarize, although the inhomogeneity of the visual field and its functional subdivisions influence visual attention effects quantitatively, especially if used stimuli are not scaled for cortical magnification, there seem to be no qualitative differences between the near and far periphery if a discrimination task is used instead of a detection task. Discrimination tasks seem to be preferable to detection tasks because they usually lead to larger cueing effects with peripheral cues and seem less influenced by the earlier onset/time course of IOR in the far periphery than detection tasks. Finally, the present study raises interesting questions for future research: (1) What are the time courses of facilitative and inhibitory cueing effects (IOR) beyond the near periphery? (2) How do these time courses vary across individuals and eccentric locations? (3) How do they interact with crowding? (4) How do detection and discrimination cueing paradigms differ beyond the near periphery?

## Figures and Tables

**Table 1:** It shows the mean and standard deviations for Cueing × Eccentricity.

Eccentricity	Valid	Neutral	Invalid	Cueing Effect
**eccentricity 15°**	512 ms (74)	570 ms (106)	560 ms (86)	48 ms
**eccentricity 30°**	579 ms (96)	593 ms (95)	596 ms (91)	17 ms
**eccentricity 56°**	629 ms (109)	683 ms (109)	701 ms (115)	72 ms

## Supplementary Materials

Data from both Experiments are available online at https://www.mdpi.com/2411-5150/4/1/13/s1, Table S1: Experiment 1; Table S2: Experiment 2.

## References

[1] Posner M.I. (1980). Orienting of attention. Q. J. Exp. Psychol..

[2] Carrasco M. (2011). Visual attention: The past 25 years. Vis. Res..

[3] Carrasco M., Nobre K., Kastner S. (2014). Spatial covert attention: Perceptual modulation. Oxford Handbook of Attention.

[4] Chica A.B., Bartolomeo P., Lupianez J. (2013). Two cognitive and neural systems for endogenous and exogeneous spatial attention. Behav. Brain Res..

[5] Moore T., Zirnsak M. (2017). Neural mechanisms of visual selective attention. Annu. Rev. Psychol..

[6] Anton-Erxleben K., Carrasco M. (2013). Attentional enhancement of spatial resolution: Linking behavioural and neurophysiological evidence. Nat. Rev. Neurosci..

[7] Bundesen C. (1990). A theory of visual attention. Psychol. Rev..

[8] Bundesen C., Habekost T. (2008). Principles of Visual Attention: Linking Mind and Brain.

[9] Schneider W.X. (2013). Selective visual processing across competition episodes: A theory of task-driven visual attention and working memory. Philos. Trans. R. Soc. B Biol. Sci..

[10] Bao Y., Lei Q., Fang Y., Tong Y., Schill K., Pöppel E., Strasburger H. (2013). Inhibition of return in the visual field. The eccentricity effect is independent of cortical magnification. Exp. Psychol..

[11] Posner M.I. (1978). Chronometric Explorations of Mind.

[12] Posner M.I., Snyder C.R.R., Davidson B.J. (1980). Attention and the detection of signals. J. Exp. Psychol. Gen..

[13] Bao Y., Pöppel E. (2007). Two spatially separated attention systems in the visual field: Evidence from inhibition of return. Cogn. Process..

[14] Bao Y., Wang Y., Pöppel E. (2012). Spatial orienting in the visual field: A unified perceptual space?. Cogn. Process..

[15] Feng J., Spence I. (2017). The effects of spatial endogenous pre-cueing across eccentricities. Front. Psychol..

[16] Casteau S., Smith D.T. (2018). Covert attention beyond the range of eye-movements: Evidence for a dissociation between exogenous and endogenous orienting. Cortex.

[17] Poggel D.A., Strasburger H., MacKeben M. (2007). Cueing attention by relative motion in the periphery of the visual field. Perception.

[18] Simpson M.J. (2017). Mini-Review: Far peripheral vision. Vis. Res..

[19] To M.S.P., Regan B.C., Wood D., Mollon J.D. (2011). Vision out of the corner of the eye. Vis. Res..

[20] Strasburger H., Rentschler I., Jüttner M. (2011). Peripheral vision and pattern recognition: A review. J. Vis..

[21] Guitton D., Volle M. (1987). Gaze-control in humans: Eye-head coordination during orienting movements to targets within and beyond the ocular motor range. J. Neurophysiol..

[22] Mikellidou K., Kurzawski J.W., Frijia F., Montanaro D., Greco V., Burr D.C., Morrone M.C. (2017). Area Prostriata in the human brain. Curr. Biol..

[23] Tamietto M., Leopold D.A. (2018). Visual cortex: The eccentric area prostriata in the human brain. Curr. Biol..

[24] Herwig A., Schneider W.X. (2014). Predicting Object Features Across Saccades: Evidence from Object Recognition and Visual Search. J. Exp. Psychol. Gen..

[25] Weiß K., Schneider W.X., Herwig A. (2014). Associating peripheral and foveal visual input across saccades: A default mode of the human visual system?. J. Vis..

[26] Weiß K., Schneider W.X., Herwig A. (2015). A “blanking effect” for surface features: Transsaccadic spatial frequency discrimination is improved by post-saccadic blanking. Atten. Percept. Psychophys..

[27] Herwig A., Weiß K., Schneider W.X. (2015). When circles become triangular: How transsaccadic predictions shape the perception of shape. Ann. N. Y. Acad. Sci..

[28] Herwig A., Weiß K., Schneider W.X. (2018). Feature prediction across eye movements is location specific and based on retinotopic coordinates. J. Vis..

[29] Bouma H. (1970). Interaction effects in parafoveal letter recognition. Nature.

[30] Land M.F., Tatler B.W. (2009). Looking and Acting: Vision and Action in Natural Behavior.

[31] Thorpe S.J., Gegenfurtner K.R., Fabre-Thorpe M., Bülthoff H.H. (2001). Detection of animals in natural images using far peripheral vision. Eur. J. Neurosci..

[32] Yeshurun Y., Rashal E. (2010). Precueing attention to the target location diminishes crowding and reduces the critical distance. J. Vis..

[33] Carrasco M., Yeshurun Y. (1998). The contribution of cover attention to set-size and eccentricity effects in visual search. J. Exp. Psychol. Hum..

[34] Golla H., Ignashchenkova A., Haarmeier T., Thier P. (2004). Improvement of visual acuity by spatial cueing: A comparative study in human and non-human primates. Vis. Res..

[35] Yeshurun Y., Carrasco M. (1999). Spatial attention improves performance in spatial resolution tasks. Vis. Res..

[36] Parsad S.G., Patil G.S., Mishra R.K. (2015). Effect of exogeneous cues on covert spatial orienting in deaf and and normal hearing individuals. PLoS ONE.

[37] Chica A.B., Martin-Arevalo E., Botta F., Lupianez J. (2014). The spatial orienting paradigm: How to design and interpret spatial attention experiments. Neurosci. Biobehav. Rev..

[38] Lou C., Lupianez J., Funes M.J., Fu X. (2013). Reduction of the stroop effect by peripheral cueing as a function of the presence/absence of placeholders. PLoS ONE.

[39] Jonides J., Mack R. (1984). On the cost and benefit of Cost and Benefit. Psychol. Bull..

[40] Posner M.I., Cohen Y. (1984). Components of visual orienting. Attention and Performance, X.

[41] Klein R.M. (2000). Inhibition of return. Trends Cogn. Sci..

[42] Danziger S., Kingstone A. (1999). Unmasking the inhibition of return phenomenon. Percept. Psychophys..

[43] Bao Y., Wang Z., Liang W., Wang Y., Pöppel E., Li H. (2013). Inhibition of Return at different eccentricities in the visual field share the same temporal window. Neurosci. Lett..

[44] Deubel H., Schneider W.X. (1996). Saccade target selection and object recognition: Evidence for a common attentional mechanism. Vision Res..

[45] Smith D.T., Rorden C., Jackson S.R. (2004). Exogenous orienting of attention depends upon the ability to execute eye movements. Curr. Biol..

[46] Smith D.T., Schenk T., Rorden C. (2012). Saccade preparation is required for exogenous attention but not endogenous attention or IOR. J. Exp. Psychol. Hum..

[47] Smith D.T., Ball K., Ellison A. (2014). Covert visual search within and beyond the effective occulomotor range. Vis. Res..

[48] Hedge C., Powell P., Sumner P. (2018). The reliability paradox: Why robust cognitive tasks do not produce reliable individual differences. Behav. Res. Methods.

[49] Rouder J.N., Haff J.M. (2019). A psychometrics of individual differences in experimental tasks. Psychon. B Rev..

[50] Lacouture Y., Cousineau D. (2008). How to use MATLAB to fit the ex-Gaussian and other probability functions to a distribution of response times. Tutor. Quant. Methods Psychol..

[51] Rizzolatti G., Riggio L., Dascola I., Umilta C. (1987). Reorienting attention across the horizontal and vertical meridians: Evidence in favor of a premotor theory of attention. Neuropsychologia.

[52] Harvey L.O., Pöppel E. (1972). Contrast sensitivity of the human retina. Am. J. Optom. Arch. Am. Acad. Optom..

[53] Harvey L.O., Pöppel E. (1973). Light-difference threshold and subjective brightness in the periphery of the visual field. Psychol. Forsch..

[54] Osaka N. (1976). Visual reaction time as a function of target size and retinal eccentricity in the peripheral visual field. Jpn. Psychol. Res..

[55] Schiefer U., Strasburger H., Becker S.T., Vonthein R., Schiller J., Dietrich T.J., Hart W. (2001). Reaction time in automated kinetic perimetry. Effects of stimulus luminance, eccentricity and movement direction. Vis. Res..

[56] Matzke D., Wagenmakers J. (2009). Psychological interpretation of the ex-Gaussian and shifted Wald parameters: A diffusion model analysis. Psychon. Bull. Rev..

